# Differences in dental arch characteristics between genders in patients with suspected condylar hyperplasia in a North Sumatra subpopulation: a cross-sectional study

**DOI:** 10.12688/f1000research.22780.3

**Published:** 2020-06-12

**Authors:** Ervina Sofyanti, Trelia Boel, Denny Satria, Zuriyah Fionita Ritonga, Indah Hafniar Hasibuan

**Affiliations:** 1 Department of Orthodontics, Faculty of Dentistry, Universitas Sumatera Utara, Medan, North Sumatra, 20155, Indonesia; 2 Department of Dentomaxillofacial radiography, Faculty of Dentistry, Universitas Sumatera Utara, Medan, North Sumatra, 20155, Indonesia; 3 Department of Pharmaceutical Biology, Faculty of Pharmacy, Universitas Sumatera Utara, Medan, North Sumatra, 20155, Indonesia

**Keywords:** dental arch, condylar hyperplasia, asymmetry, form

## Abstract

**Background:** Condylar Hyperplasia (CH) is a self-limiting mandibular condyle disorder that shows asymmetry progress in conjunction with associated occlusal changes as long as condylar growth is still active and leads to facial asymmetry. This study aimed to analysis the difference of dental arch characteristics  based on genders in orthodontic patients with suspected CH in a North Sumatra subpopulation.

**Methods:** This is a retrospective study of suspected CH patient’s clinical records who sought for the initial orthodontic treatment between January 2015 to March 2019. Patient with facial asymmetry (based on photography, posterior crossbite and midline deviation), the positive temporomandibular joint disorder in functional analysis, and no history of facial trauma were included in the study. Dental arch asymmetry was based on the measurement of dental midline deviation, canine tip in the dental arch, the distance of the upper canines from the palatal suture, and inter canine distance. The evaluation of dental arch was achieved by comparing arch width and length.

**Results:** There was a significant difference (p<0.05) of upper canine distance from the palatal suture in female patients when evaluating upper dental arch asymmetry. There was a moderate correlation (r=0.379) in midline deviation between upper and lower dental arch. The dimension and dental arch form were mid and flat, and there was moderate correlation (r=0.448) between the upper and lower dental arch form in these suspected CH patients.

**Conclusion:** Indeed skeletal asymmetry, the evaluation of the dental arch characteristic symmetry and arch form showed asymmetric occlusal characteristics in orthodontics patient with suspected  CH in the North Sumatera subpopulation. In treating these patients, we recommend the plaster cast evaluation as essential and routine procedure in order to understand the complexity of occlusal change due to active growth of condylar and limitation in radiography evaluation.

## Introduction

The unilateral non-neoplastic overgrowth of mandibular condyle that leads to occlusal interferences and joint dysfunction or pain, is identified as condylar hyperplasia (CH). The elongation of condylar head, neck, and lower arch corpus leads to the development of occlusal disharmony, dental compensation, and asymmetry due to continued abnormal growth that can be unilaterally or bilaterally ( Bharathi *et al.*, 2014; Obwegeser & Makek, 1986; Wolford *et al.*, 2014). This deformity requires assessment in condylar growth and sometimes requires high condylectomy combined with orthognathic surgery and articular disc repositioning to achieve treatment stability ( Lippold *et al.*, 2007; Wolford *et al.*, 2009). Sequential assessments (six to twelve month intervals) of CH aim to prevent worsening functional, esthetic, skeletal, and occlusal changes during orthodontic treatment. These examinations include clinical examinations, plaster cast analysis, and radiographic evaluations in the abnormal condylar lower arch growth. Since CH is a rare disorder, previous studies explore the multiple factors of mandibular growth asymmetry and development. The characteristics of CH are as follows: excessive or persistent growth of condyle leads to facial asymmetry with occlusal discrepancies and temporomandibular joint disorder as concurrent symptoms. It has severe asymmetry over a long-time period and can grow past the growth period gradually ( Almeida *et al.*, 2015; Goulart *et al.*, 2018; Rajimakers *et al.*, 2012; Wolford *et al.*, 2014). The CH treatment modality is differ depending on affected structures, patient’s age, severity of asymmetry, and the activity status of CH. ( Lopez & Corral, 2015; Obwegeser & Makek, 1986; Wolford *et al.*, 2014).

Previous studies report several types of CH, based on radiographic and clinical characteristics that related to enlargement or hyperplasia of mandibular that will help the dental clinicians in understanding the complexity of facial asymmetry in 3 dimension ( López *et al.*, 2019; Obwegeser & Makek, 1986; Wolford *et al.*, 2014). CH usually occurs during puberty and ceases at the same time as the completion of healthy growth ( Rajimakers *et al.*, 2012; Wolford *et al.*, 2014). The types of CH including hemimandibular hyperplasia as condylar asymmetry in vertical direction, hemimandibular elongation as condylar asymmetry in horizontal direction, and hybrid ( Obwegeser & Makek, 1986; Wolford *et al.*, 2014). The similar symptoms of glenoid fossa asymmetry and hemimandibular elongation that led to the difference in therapeutic approach and suggested for careful correlation of clinical findings and imaging tests ( Lopez & Botero, 2017). CH affects women more than men with a 3:1 ratio ( Nitzan *et al.*, 2008). It can also continue growing past the growth period ( Almeida *et al.*, 2015; Obwegeser & Makek, 1986; Pacheco *et al.*, 2010). The investigation of facial asymmetry with CH complexity should be integrally planned during orthodontic treatment ( Olate *et al.*, 2013). Previous report cases mentioned that condylectomy to stop CH, surgery to correct skeletal discrepancy, and facelifts, augmentation using medpor implants or fat grafts to achieve aesthetics in treatment objectives ( Alyamani & Abuzinada, 2012; Lopez & Herrera-Guardiola, 2016; López *et al.*, 2017).

Previous studies report that CH adversely affects morphology and size of the mandible, it also alters the occlusion development along with dental arches ( Goulart *et al.*, 2018; Mehrotra *et al.*, 2011; Wolford *et al.*, 2014). According to Purbiati’s study in Indonesian sub-population student between 8 to 30 year old, male subject had a higher risk of mandibular asymmetry occurance 2.447 times than female ( Purbiati *et al.*, 2016) In planning orthodontics treatment, changing the dental arch form can also influence treatment stability. In patients with facial asymmetry, dental alveolar compensation is one treatment choice when treating malocclusion without surgery intervention. Due to the the genetic and enviromental factors influence occlusal development and limitation of radiography evaluation this study aims to analysis the dental arch asymmetry and form in pre-orthodontic patients with suspected CH based on genders

## Methods

This is a retrospective study of the clinical records of suspected CH patients that sought for the initial orthodontic treatment at the Dental Hospital, Universitas Sumatra Utara between January 2015 and March 2019. The Research Ethics Committee of the Universitas Sumatera Utara Medical Faculty approved this study (378/TGL/KEPK FK USU-RSUP HAM/2019). Those patients who were treated with fixed orthodontics at the Dental Hospital, University of Sumatra have signed informed consent, which includes data collection for research purposes.

### Participants

CH suspected subjects fulfilled the following characteristics: facial asymmetry based on photography, posterior cross bite and midline deviation in plaster casts analysis, and positive temporomandibular joint disorder in functional analysis from the dental records. Since this is a retrospective study of suspected CH patient’s clinical records, we included the subjects with pain, clicking, crepitation, and other signs or symptoms of TMD from extra oral analysis in their dental record. This study excluded patients with a history of facial trauma and hereditary disorder based on their medical records, and patients whose records had unclear morphology of condylar and lower arch features in pretreatment panoramic radiographs so that cannot be analyzed with asymmetry vertical mandibular with Kjellberg’s technique ( Hirpara *et al.*, 2016).

### Data analysis

Evaluation of dental arch symmetry was done using plaster casts taken from the patients’ dental records.

***Initial measurements.*** The well-trimmed plaster casts were positioned on millimeter rule paper and measurements were taken manually with a cephalometric and metal protractor. To determine the upper arch midline, a mark was located along the mid palatal suture. The symmetry axis of the upper arch midline was made by connecting the incisive papilla (As=Anterior superior) passing second palatal rugae to the most visible posterior upper arch landmark (Ps=Posterior superior) over the mid palatal suture. Once the Ps was marked, the end reference point was located on the border between the hard and soft palate. Then Ps mark of the upper arch was transferred to the lower arch (Pi=Posterior inferior) using a ruler positioned perpendicular and occluded upper and lower plaster casts. To determine the anterior lower arch point (Ai=Anterior inferior), the upper arch As was transferred to the lower arch using a ruler that occluded upper and lower plaster casts. The symmetry axis of the lower arch midline was obtained by connecting the Ai to the most visible posterior landmark (Pi) over the lingual frenulum to the posterior border of lower arch. After marking the midline symmetry axis, we can evaluate the dental arch asymmetry by measuring dental midline deviation (MD), canine tip in the dental arch (PC), distance of the upper canines from the palatal suture (DC) and inter canine distance (ICD) in the upper and lower arches ( Scanavini *et al.*, 2012).

**Figure 1.**
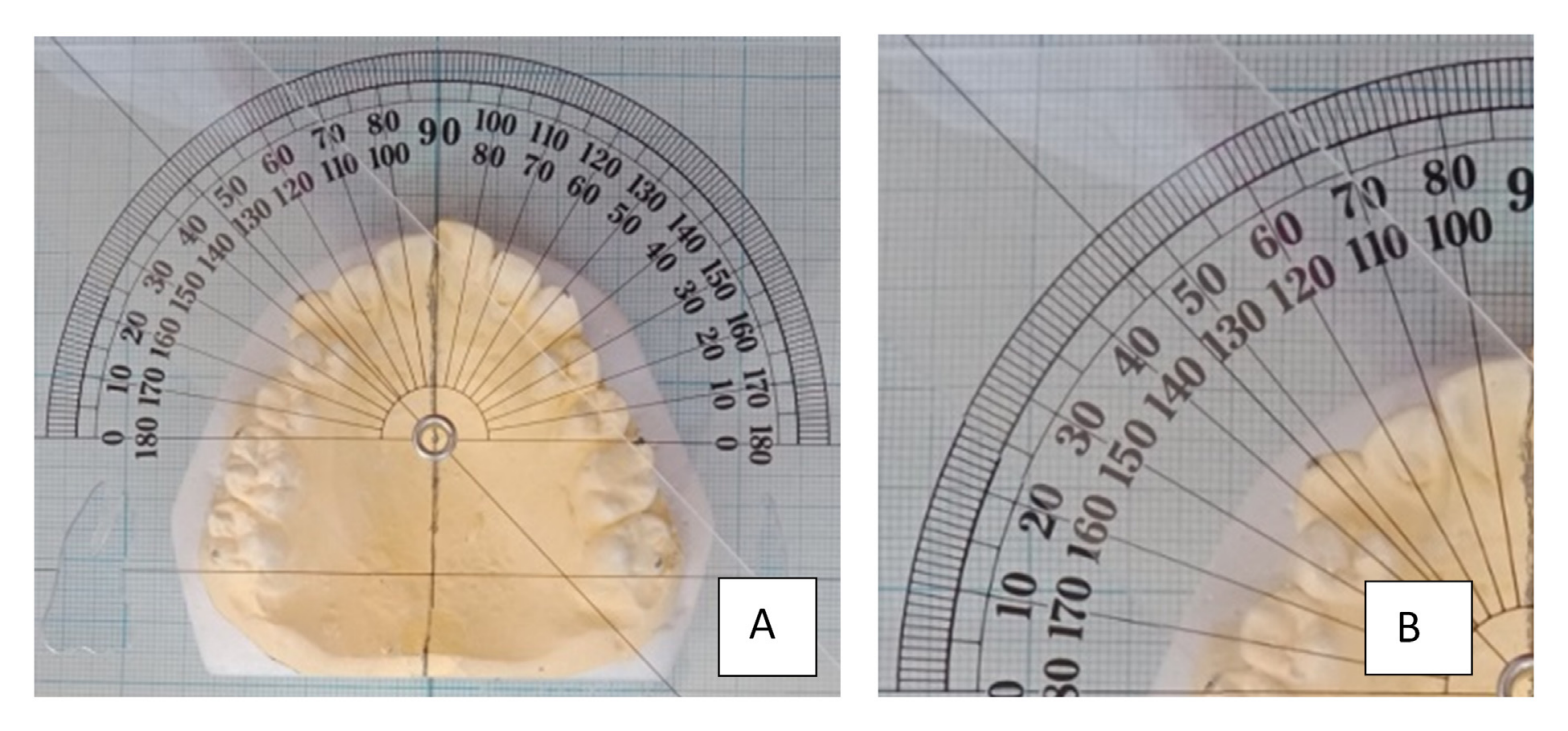
Measuring angular upper arch asymmetry. The plaster cast model is positioned with a protractor at the cusp of the right upper arch canine ( **A**); the protractor shows that PC of right upper arch canine is 46°±1° ( **B**).

**Figure 2.**
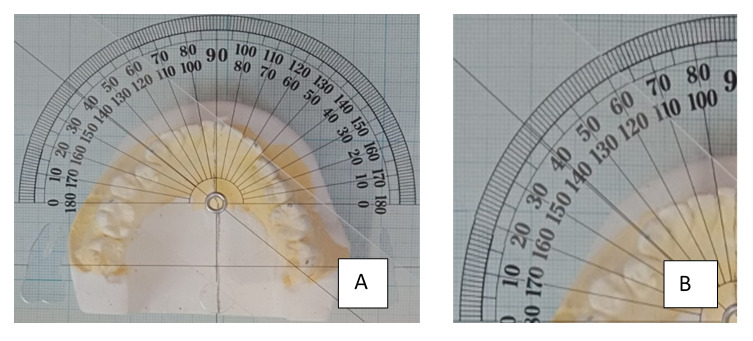
Measuring angular lower arch asymmetry. The plaster cast model is positioned with a protractor at the cusp of the right lower canine ( **A**); the protractor shows that PC of right lower arch canine is 39°±1° ( **B**).

**Figure 3.**
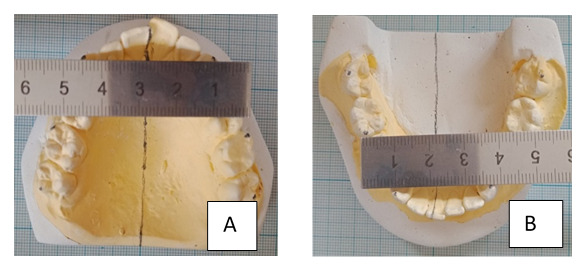
Measuring distance of the canines in upper and lower arch asymmetry.

***Angular and linear measurement of the dental arch asymmetry.*** Angular arch asymmetry was performed by measuring the right and left canines to the upper arch symmetry axis using a cephalometric protractor in the upper arch (this is known as midline deviation (MD) ( Figure 1 and Figure 2). The position of the canine (PC) is the distance from the cusp tip on each side that were projected perpendicular to the symmetry axis using a metal protractor. Distance of canines (DC) is the linear measurement from right to left canine tips ( Figure 3). This angular and linear measurement of upper and lower arch dental asymmetry has predictive accuracy and is marked as negative if shifted to the left ( Scanavini *et al.*, 2012).

**Figure 4.**
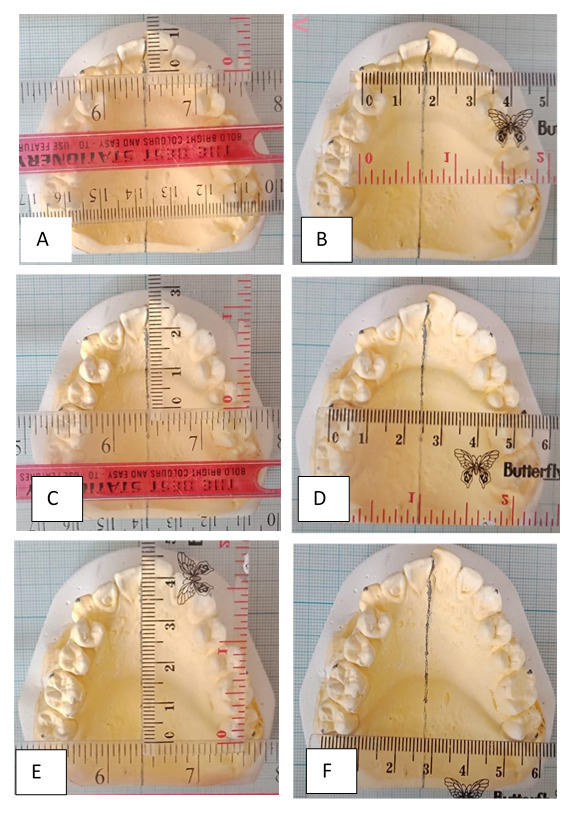
Measurement of upper dental arch form. The sagittal dental arch from the arrow of the anterior curve (L31) ( **A**); from right canine cusp to left canine cusp is identified as intercanine width (L33) ( **B**); the incisal edge to the line joining the mesiobuccal canines of the first molar (L61) ( **C**); the dental arch dimension from right first molar cusp to the left first molar cusp is intermolar width (L66) ( **D**); the incisal edge to the line joining the distobuccal cusp of the second molar (L71) ( **E**); from right second molar cusp to the left second molar cusp is the posterior intermolar width (L77) ( **F**).

**Figure 5.**
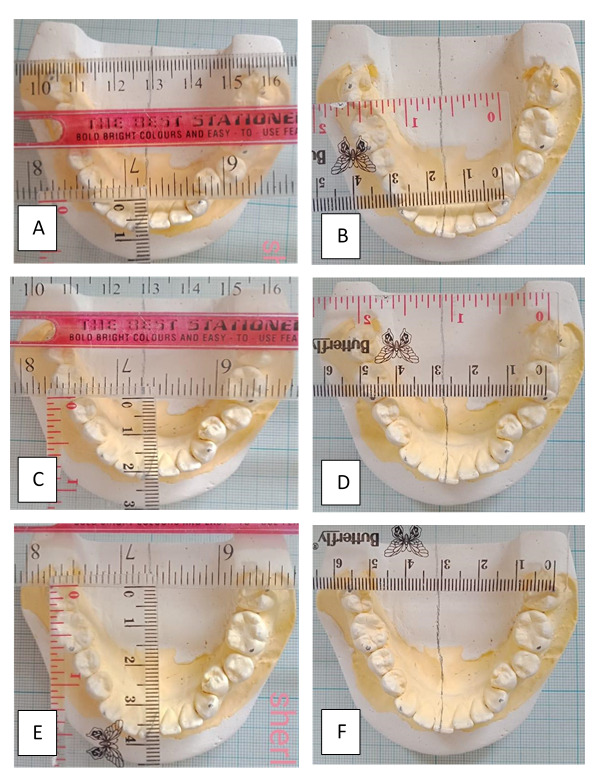
Measurement of lower dental arch form. The sagittal dental arch from the arrow of the anterior curve (L31) ( **A**); from right canine cusp to left canine cusp is identified as intercanine width (L33) ( **B**); the incisal edge to the line joining the mesiobuccal canines of the first molar (L61) ( **C**); the dental arch dimension from right first molar cusp to the left first molar cusp is intermolar width (L66) ( **D**); the incisal edge to the line joining the distobuccal cusp of the second molar (L71) ( **E**); from right second molar cusp to the left second molar cusp is the posterior intermolar width (L77) ( **F**).

***Evaluation of dental arch form.*** The evaluation of dental arch form by comparing arch width and length. Dental arch dimensions were as follows: L33 (inter canine width), from right canine cusp to left canine cusp; L66 (intermolar width), from right first molar cusp to left first molar cusp; L77 (posterior intermolar width), from right second molar cusp to left second molar cusp; L31 (sagittal dental arch), from the “arrow” of the anterior curve; L61, from the incisal edge to the line joining the mesiobuccal canines of the first molar; L71, the incisal edge to the line joining the distobuccal cusps of the second molar. These six factors characterized both the arch form and the dimension that will determine the dental arch ratio: L31/L33, L61/L66, L71/L77, L33/L66, L61/L71 ( Figure 4 and Figure 5). The arch is classified as narrow if three dental arch ratios (L31/L33, L61/L66, L71/L77) are positive, the arch form is classified as wide if they are negative. The arch is classified as mid if none of those dental arch ratios significantly deviates from the average. The arch form is pointed if only the L31/L33 has a noticeable intensity significantly higher value than all the comparisons (L61/L66, L71/L77, L33/L66, L61/L71). The arch is classified as flat only if the L31/L33 has significantly lower value than all comparisons (L61/L66, L71/L77, L33/L66, L61/L71) ( Raberin *et al.,* 1993). Those measurements were repeated in 15 days after obtaining the initial data to assess the reliability with intra-rater measurement.

***Statistical analysis.*** Analyzing the significant difference of symmetrical angular and linear measurement of the dental arches based on gender was done using *paired independent pair t-test* (p<0.05). This study used the second measurement in analyzing symmetrical dental and dental arch form with *chi-square* and *Cramer’s V correlation.* SPSS version 17.0 was used for data analysis.

## Results

There were total of 14 male and 20 female patients who were suspected as CH patients between 18 to 27 years old based on date of birth stated in the medical record. Those subjects with suspected CH fulfilled some criterias, including:facial asymmetry based on photography analysis, positive temporomandibular joint disorder, vertical mandibular asymmetry based on pre treatment panoramic radiographs, and posterior cross bite. Table 1 shows the distribution of upper and lower arch dental symmetry in this study. Based on gender, there was no significant difference in upper and lower dental symmetry between male and female ( Table 2). There was a significant difference (p<0.05) of upper canine distance from the palatal suture in female patients, but no significant difference in other symmetry variables between male and female ( Table 3), There was also no significant difference of midline deviation between male and female in this study ( Table 4). However, there was a moderate correlation (r=0.379) of midline deviation in upper and lower dental arches of this studied subjects ( Table 5). In these CH patients, the dental arch form in upper and lower dental arch were mid and flat. There was also a moderate correlation (r=0.448) between upper and lower dental arch form ( Table 6).

**Table 1.** Distribution of upper and lower dental arch symmetry in condylar hyperplasia patients.

	Variable	Min ( *)	Max ( *)	Mean±SD ( *)
**Upper dental arch**	MD (°)	-8	7	-0.765±2.544
	PC Right (°)	30	52	40.250±4.747
	PC Left (mm)	26	54	40.941±5.585
	DC Right (mm)	8	23	16.794±2.253
	DC Left (mm)	12	20	17.559±1.673
**Lower dental arch**	MD (°)	-12	10	-1.750±5.207
	PC Right (°)	24	49.5	32.794±5.282
	PC Left (°)	24	54	36.147±6.762
	DC Right (mm)	9	18	13.147±2.155
	DC Left (mm)	10	18	14.088±2.002

*Negative, the midline deviates to the left-side; positive, deviates to the right-side. MD : Midline Deviation PC : Canine tip in the dental arch DC : Distance of upper canine from the palatal suture

**Table 2.** Difference of angular and linear measurement for symmetrical analysis in upper and lower dental arch based on gender.

	Variable	Gender	N	Mean±SD ( *)	*p*
**Upper dental arch**	MD(°)	Male	14	0.036±2.707	0.358
		Female	20	-1.325±2.330	
	Right PC(°)	Male	14	38.750±4.004	0.150
		Female	20	41.300±5.035	
	Left PC(°)	Male	14	39.607±5.115	0.232
		Female	20	41.875±5.835	
	Right DC(mm)	Male	14	17.536±2.098	0.228
		Female	20	16.275±2.262	
	Left DC(mm)	Male	14	17.393±2.021	0.943
		Female	20	17.675±1.426	
**Lower dental arch**	MD(°)	Male	14	-1.250±4.154	0.549
		Female	20	-2.100±5.913	
	Right PC(°)	Male	14	32.821±6.043	0.944
		Female	20	32.775±4.844	
	Left PC(°)	Male	14	35.214±7.645	0.318
		Female	20	36.800±6.191	
	Right DC(mm)	Male	14	13.036±2.231	0.711
		Female	20	13.225±2.155	
	Left DC(mm)	Male	14	13.714±2.128	0.492
		Female	20	14.350±1.920	

*Negative, the midline deviates to the left-side; positive, deviates to the right-side. Statistically significant difference ( *p*<0.05). MD : Midline Deviation PC : Canine tip in the dental arch DC : Distance of upper canine from the palatal suture

**Table 3.** Difference of symmetry variables of upper and lower dental arch based on gender.

	Sex	Variable	Side	Mean	*p*
**Upper dental arch**	Male	PC(°)	Right	38.75±4.00	0.185
			Left	39.61±5.11	
		DC (mm)	Right	17.53±2.10	0.672
			Left	17.40±2.02	
	Female	PC(°)	Right	41.30±5.04	0.534
			Left	41.87±5.84	
		DC (mm)	Right	16.28±2.26	0.010*
			Left	17.68±1.43	
**Lower dental arch**	Male	PC(°)	Right	32.82±6.04	0.551
			Left	35.21±7.65	
		DC (mm)	Right	13.04±2.23	0.449
			Left	13.71±2.13	
	Female	PC(°)	Right	32,78±4.84	0.126
			Left	36.80±6.20	
		DC (mm)	Right	13.23±2.16	0.102
			Left	14.35±1.92	

Statistically significant difference (p<0.05). PC : Canine tip in the dental arch DC : Distance of upper canine from the palatal suture

**Table 4.** Difference of midline deviation based on gender in condylar hyperplasia patients.

Midline deviation		Male, n (%)	Female, n (%)	*p*
**Upper dental arch**	Normal	6 (42.9)	9 (45)	0.429
	Shifted to the right	3 (21.4)	1 (5)	
	Shifted to the left	5 (35.7)	10 (50)	
**Lower dental arch**	Normal	1 (7.1)	4 (20)	0.512
	Shifted to the right	6 (42.9)	6 (30)	
	Shifted to the left	7 (50)	10 (50)	

Statistically significant difference ( *p*<0.05).

**Table 5.** Correlation of midline deviation in upper and lower dental arch in in condylar hyperplasia patients.

Dental Arch Asymmetry		Lower arch			*p*	*r*
		Normal	Shifted to the right	Shifted to the left		
Upper arch	Normal	3	8	4	0.039*	0.379
		20.0%	53.3%	26.7%		
	Shifted to the right	1	2	1		
		25.0%	50.0%	25.0%		
	Shifted to the left	1	2	12		
		6.7%	13.3%	80.0%		

Statistically significant difference ( *p*<0.05).

**Table 6.** Correlation of upper and lower dental arch form in condylar hyperplasia patients.

Dental Arch Form		Lower arch		*p*	*r*
		Mid	Flat		
Upper arch	Mid	9	4	0.014*	0.448
		69.2%	30.8%		
	Flat	5	6		
		23.8%	76.2%		

Statistically significant difference ( *p*<0.05).

## Discussion

The dental arch evaluation is an essential issue in orthodontic treatment. We performed a retrospective study of clinical records of suspected CH patients in order to evaluate dental arch symmetry. The failure in early identification of CH can lead to unfavorable esthetic and functional orthodontic treatment resulting in the development of asymmetry ( Almeida *et al.*, 2015; Lopez & Corral, 2015). Identifying the growth pattern occurring in CH and deciding the activity of condylar growth are important in treating any malocclusion with lower arch asymmetry. Wolford *et al.* introduced an updated classification system based on growth, clinical, histological, and imaging characteristics ( Wolford *et al.*, 2014). Progressive unilateral growth expansion of condyle, leads to facial asymmetry, malocclusion, and indirectly affects the upper arch development, also shifting off the chin towards the unaffected side ( Goulart *et al.*, 2018; Wolford *et al.*, 2014). A study by Mehrotra *et al.* in 2011 on five Indian adult active CH patients reported that facial asymmetry was the most common clinical finding, followed by midline shifting, protruded chin, contralateral cross bite, and upper arch occlusal canting ( Mehrotra *et al.*, 2011). CH clinical findings show midline shifting along with chin deviation to contralateral side with related posterior cross bite ( Almeida *et al.*, 2015). Generally, patients with active CH present facial asymmetry transversally, vertically or combined ( Nitzan *et al.*, 2008). There was a significant difference in soft-tissue asymmetry between CH subjects and controls as observed in three-dimensional photographs which can be used in evaluation of progressive worsening occlusion while radiography is limited ( Verhoeven *et al.*, 2013).

Most studies have also reported that CH occurs between ages 10 and 30 years with abnormal growth of the hyperplasia cases and the hemimandibular hyperplasia occurs at significantly younger age ( Bharathi *et al.*, 2014; Goulart *et al.*, 2018; Lopez & Herrera-Guadiola., 2016; López *et al.*, 2017; Pacheco *et al.*, 2010). The 3-dimensional radiographs and quantitative techniques are useful for evaluation, classification, or follow up of CH although the emergence of panoramic radiographs might be suitable for screening CH in some emerging countries. ( Nolte *et al.*, 2015). There were no significant association between vertical mandibular asymmetry and temporomandibular disorder symptom ( Mendoza-García *et al.*, 2019; Sofyanti *et al.*, 2018a) whilst there was a specific pattern of dental and skeletal morphology with temporomandibular disorder sign in Indonesian subpopulation between 8 to 30 year old. The absence of 3D diagnostic radiography in this study encountered photography, positive posterior cross bite from plaster cast, and positive temporomandibular disorder in a cohort of 18 to 27 year old patients based on initial dental record.

Table 1 shows that in our patients the midline shifted to the left on the upper and lower arch. This result might be supported by Haraguchi *et al*’s study that reported a higher proportion of wider left hemiface in the post pubertal stage than prepubertal stage in 1800 Japanese subjects ( Haraguchi *et al.*, 2008). However, this study result was different to a clinical study in Brazilian twin girls with CH who presented with facial asymmetry and skeletal class III whose chin and lower arch midline tend to deviate to the right side ( Goulart *et al.*, 2018).

Even though previous study reported that CH affects more women than men, with a 3:1 ratio ( Nitzan *et al.*, 2008); however, there was no significant difference of angular and linear measurement for symmetrical analysis in upper and lower dental arch based on gender in this study ( Table 2) that similar with Rajimaker’s study that there was no evidence that the side affected by CH was linked to sex ( Rajimakers *et al.*, 2012). There was also no significant difference of symmetry variables in upper and lower arch dental arch of CH patients based on gender, except the upper canines distance from the palatal suture of female patients ( Table 3) that might be related to the anatomical characteristics of female arch rather than hyperplasia process. While this study included the positive temporomandibular joint disorder as a concurrent symptoms with facial asymmetry, this dysfunction is not a pathognomonic sign of the pathology ( López *et al.*, 2019) and might not always correlated to CH as well as posterior cross bite and midline deviation ( Purbiati *et al.*, 2016). The outcome of this study was also suggested by Al-Zubair, which reported that the location of the central incisor and canines to each other and to other teeth is the most potent factor in determining dental arch asymmetry ( Al-Zubair, 2019). Thus, the characteristics of brackets should be well understood by the clinician to obtain the desired teeth movement in achieve proper dental arch, correct angle classification or torque of the posterior teeth in CH treatment ( Lopez & Herrera-Guardiola, 2016).

According to Scanavini *et al,* the differences of midline deviation in normal and class II malocclusion, is midline deviation dominant toward the left in the upper arch in each group ( Scanavini *et al.*, 2012). In this study, the different PC between the upper canines distance from the palatal suture were higher in the lower arch than the upper arch. As with the analysis of midline deviation, the lower arch showed a higher degree of asymmetry than its counterpart upper arch. In the current study, the average values of midline deviation were greater than in the previous study regardless of the type of the malocclusion. Since this study focused on CH patients, the variance of symmetry showed a moderate correlation in midline deviation of dental arch symmetry ( Table 4) and dental arch form ( Table 5). The presence of condylar hyperactivity influenced the degree of malocclusion with facial asymmetry and diversity of CH ( Alyamani & Abuzinada, 2012; Lopez & Corral, 2015; Pacheco *et al.*, 2010; Pinto *et al.*, 2016).

Prasad *et al.* reported that dental arch width of untreated South Indian adults which had minimal crowding and spacing, is associated with gender, race and vertical facial morphology. Then, it was important to consider the individualized arch wires ( Prasad *et al.*, 2013) In Saudi normal population, it is suggested using a nickel titanium arch wire that will produce the lowest mean absolute error, cause minimal change in the dental arch form, and less customization of stainless steel wires necessary ( Al-Barakati *et al.*, 2016). If there was a failure to preserve the arch form, it might increase the probability of relapse ( Bayome *et al.*, 2011). Relapse related to improper arch wire changes can affect periodontal breakdown, recurrence of crowding of buccal segments, or increased crowding of labial segments particularly while inter-canine width and inter-molar width have been expanded. In the present study, relatively stocky arch guide showed mid and flat arch form for both upper and lower dental arch in the studied patients. This is also similar to the lower dental arch form of a Papuan population, which showed that prevalence of mid arch form was highest whilst the pointed lower arch form was the least in lower arch analysis of 18 to 25 year old Papuan students in Manado ( Saputra *et al.*, 2016). Another Indonesian sub-population study also reported that there was only significant difference of upper dental arch for full dentition of Buginese, Makassarese, and Torajanese individuals, which was null in the lower dental arch ( Rieuwpassa *et al.*, 2012).

In interceptive orthodontic treatment, dental arch coordination should be evaluated during occlusal changing during mandibular growth. The development asymmetry of the lower arch might be due to the condylar growth activity and joint function and it is common that a growth period elapses after the patient’ asymmetric growth is completed ( Pacheco *et al.*, 2010). In order to provide the optimal orthodontic treatment, Lopez’ studies suggested that the differential diagnosis of CH requires overwhelmed understanding of aetiology until differences therapeutic approach based on clinical findings, radiography analysis, and histological characteristics of condylar hyperactivity. ( Lopez & Corral, 2015; López *et al.*, 2017)

The asymmetry upper dental arch showed highly significant difference between right and left posterior arch segment whilst anterior segment showed no significant difference in class I occlusion of Iraqi young adult ( Allabban *et al.*, 2017). There was significantly larger condylar length and the condylar sum of maximum values in the affected side and also positive correlation between the increased dimensions of the articular eminence and the more posterior position of the glenoid fossa in suspected Colombian CH patients ( Lopez & Botero, 2020). Further study, such as case-control study, genetic investigation, histology findings and follow-up of the treatment progress may help clinicians to understand the multi-characteristics of CH among races. Ethnicity also influences the development of dental arch form related with symmetry point. Genetic and environmental factors can influence the development of left-right patterning during embryogenesis and remain in the masseter muscle in adults, including the *PITX2* promoter ( Sofyanti *et al.*, 2018b). In the future, evaluation of plaster casts during orthodontic treatment is a requirement especially in treating malocclusion with mandibular asymmetry in order to anticipate the biomechanics of certain arch wire type. The clinicians should be careful in using arch wire coordination to obtain optimal occlusal relationship in order to maintain treatment stability while treating orthodontic patients with suspected CH due to dentoalveolar compensation of asymmetric growth ( Al-Barakati *et al.*, 2016; Bayome *et al.*, 2011; Lopez & Herrera-Guardiola, 2016; Prasad *et al.*, 2013).

## Conclusion

Indeed skeletal asymmetry, the evaluation of the dental arch characteristic symmetry and arch form showed asymmetric occlusal characteristics in orthodontics patient with suspected. However, there was no significant differences of dental arch characteristic based on genders in these studied subjects. In treating these patients, plaster cast evaluation is recommended as essential and routine procedure in order to understand the complexity of occlusal characteristics due to active growth of condylar and limitations in radiography evaluation during treatment. This phenomenon is marked as one obstacle in treating growing patients while CH is not detected early.

## Data availability

Harvard Dataverse: Replication Data for: Angular and linear measurement of the dental arch asymmetry and dental arch form, https://doi.org/10.7910/DVN/GONORA ( Sofyanti *et al.*, 2020).

Data are available under the terms of the Creative Commons Zero "No rights reserved" data waiver (CC0 1.0 Public domain dedication).
