# Supplementary material for: Differences in dental arch characteristics between genders in patients with suspected condylar hyperplasia in a North Sumatra subpopulation: a cross-sectional study
Source: F1000Res. 2020 Jun 12;9:263. Originally published 2020 Apr 16. [Version 3] doi: 10.12688/f1000research.22780.3 (PMC7295084; doi:10.12688/f1000research.22780.3)
Supplement: Peer review history

# Peer review: Reviewer response for version 3

**DOI:** 10.5256/f1000research.27046.r64676

**Published:** 2020-06-22

**Authors:** Diego Fernando López

**Affiliations:** 1 Departamento de Ortodoncia, Universidad del Valle, Cali, Colombia

The authors have adequately followed the recommendations made and those that cannot be corrected due to the nature of the study have been presented as limitations of the study in the discussion section.

Is the work clearly and accurately presented and does it cite the current literature?

Partly

If applicable, is the statistical analysis and its interpretation appropriate?

Yes

Are all the source data underlying the results available to ensure full reproducibility?

Partly

Is the study design appropriate and is the work technically sound?

No

Are the conclusions drawn adequately supported by the results?

Partly

Are sufficient details of methods and analysis provided to allow replication by others?

No

Reviewer Expertise:

Orthodontics, condylar hyperplasia, facial asymmetry.

I confirm that I have read this submission and believe that I have an appropriate level of expertise to confirm that it is of an acceptable scientific standard.

# Peer review: Reviewer response for version 2

**DOI:** 10.5256/f1000research.26748.r63485

**Published:** 2020-06-01

**Authors:** Maria Purbiati

**Affiliations:** 1 Department of Orthodontics, Faculty of Dentistry, Universitas Indonesia, Jakarta, Indonesia

**Introduction:**

 Some literature stated that CH could be performed bilaterally even though mostly unilateral. It will be more clear if the terminology is used as unilateral CH or CH. The terminology of CH only is referring to the research subjects including both unilateral and bilateral overgrowth. It should be stated consistently from the beginning (from the title). 

**Methods:**

 Too many criteria in determining facial asymmetry which will generate heterogeneous samples that lead to wide variation of samples. It seems the aim of the study to evaluate the dental arch in facial asymmetry subjects, thus extra oral (photography) or skeletal (PA cephalometric) parameter will be enough to differentiate a facial asymmetry. Then TMJ assessment was not correlated to this as well as dental record (posterior crossbite and midline deviation) which in some cases were not inline with the facial or skeletal asymmetry. 

**Data analysis:**

 Data analysis means statistical data analysis. Measurements should be explained as research methods.

 Measuring a 3-dimensional object will be more accurate by laser or digital device, such as Coordinate Measuring Machine.

**Result:**

 Wide range of age including growing and non-growing patients. Asymmetry is a result of growth and development. Dividing into two groups will give deeper analysis.

 Asymmetry is a qualitatively relative comparison between right and left side. This study's aims seem to describe dental arches condition in a facial asymmetry due to CH. So that, the amount of the measurement itself was not really important information. For this instance, categorical data (symmetrical and asymmetrical) would serve a better analysis than quantitative data, and would be emphasizing the intended context consistently,thus developing a sharp conclusion. Otherwise it needed control group for quantitative comparison.

Is the work clearly and accurately presented and does it cite the current literature?

Yes

If applicable, is the statistical analysis and its interpretation appropriate?

Partly

Are all the source data underlying the results available to ensure full reproducibility?

Yes

Is the study design appropriate and is the work technically sound?

Yes

Are the conclusions drawn adequately supported by the results?

Yes

Are sufficient details of methods and analysis provided to allow replication by others?

Partly

Reviewer Expertise:

Orthodontics, Facial Aesthetics, Asymmetry, 3 dimension imaging, Orthognathic surgery

I confirm that I have read this submission and believe that I have an appropriate level of expertise to confirm that it is of an acceptable scientific standard.

## References

[1] : Prediction of Mandibulofacial Asymmetry using Risk Factor Index and Model of Dentocraniofacial Morphological Pattern. *Journal of International Dental and Medical Research*.2016;9(3) :195-201 Reference source

# Response

**Published:** 2020-06-05

**Authors:** Ervina Sofyanti

**Affiliations:** Department of Orthodontics, Faculty of Dentistry, Indonesia

Dear Dr. Maria Purbiati,

Thank you very much for your kind assistance in reviewing our manuscript. We have revised some sections as follows:

- **Introduction**

**Some literature stated that CH could be performed bilaterally even though mostly unilateral. It will be more clear if the terminology is used as unilateral CH or CH. The terminology of CH only is referring to the research subjects including both unilateral and bilateral overgrowth. It should be stated consistently from the beginning (from the title).**

We thank you for the suggestion. We have added the information in the introduction section. However, we cannot state consistently from the beginning due to suspected CH and the limited of radiograph that supposed to be 3D CBCT as the gold standard diagnostic.

 

- **Methods:**

**Too many criteria in determining facial asymmetry which will generate heterogeneous samples that lead to a wide variety of samples. It seems the aim of the study to evaluate the dental arch in facial asymmetry subjects, thus extraoral (photography) or skeletal (PA cephalometric) parameter will be enough to differentiate facial asymmetry. Then TMJ assessment was not correlated to this as well as a dental record (posterior crossbite and midline deviation) which in some cases were not in line with the facial or skeletal asymmetry.**

We thank you for the comment. Based on the previous study, CH is a self-limiting pathology in the condylar head that affecting facial symmetry and occlusion and may be associated with pain and dysfunction. Therefore we try to make a selection case base on those criteria without 3D radiography that supposed to be the gold standard.

 

- **Data analysis:**

**Data analysis means statistical data analysis. Measurements should be explained as research methods. Measuring a 3-dimensional object will be more accurate by laser or digital device, such as Coordinate Measuring Machine**.

We thank you for the kind suggestion. Due to the certain limitation of radiography and since this was a retrospective study, we will revise in the future study. We have added the statement in the discussions as followed: The 3-dimensional radiographs and quantitative techniques are useful for evaluation, classification, or follow up of CH although the emergence of panoramic radiographs might be suitable for screening CH in some emerging countries.

 

- **Result:**

**Wide range of age including growing and non-growing patients. Asymmetry is a result of growth and development. Dividing into two groups will give a deeper analysis.**

Thank you for the kind review. Since we know that age is a factor which should be considered in determining the treatment modality, we have limited the age of sample from 10 to 30 year old based on previous studies about CH. Further study will require more samples in order to make a classification based on a growing pattern.

**Asymmetry is a qualitatively relative comparison between the right and left sides. This study's aims seem to describe the dental arch's condition in facial asymmetry due to CH. So, the amount of measurement itself was not really important information. For this instance, categorical data (symmetrical and asymmetrical) would serve a better analysis than quantitative data and would be emphasizing the intended context consistently, thus developing a sharp conclusion. Otherwise, it needed a control group for quantitative comparison.**

Thank you for your kind advice. We will consider the control group in future studies. As additional information, we compare the result with a subpopulation of Indonesian that aged between 8 to 30-year old based on Purbiati’s study.

# Peer review: Reviewer response for version 2

**DOI:** 10.5256/f1000research.26748.r63488

**Published:** 2020-05-26

**Authors:** Diego Fernando López

**Affiliations:** 1 Departamento de Ortodoncia, Universidad del Valle, Cali, Colombia

- The authors make an investigation in which they observe characteristics in the dental arches of patients with suspected condylar hyperplasia (HC). Since they do not use a group of patients without suspected HC as a control group, they can only provide descriptive statistics and the only comparison they can make is between genders or according to age. Therefore the title should mention "differences in dental arch characteristics between genders in patients with suspected HC".
- With respect to materials and methods, the authors use a millimeter rule to take measurements. There are now digital micrometric gauges as well as digital compasses to establish more accurate measurements that deliver decimals and thus provide measurements that may lead to statistically significant differences. (Measurements should be taken with these instruments that provide better accuracy). 
- Additionally, the overlap of the upper palatal midline over the lower arch may be very inaccurate with manual techniques. At present, scanned models, tomography and the use of software reduce these errors in the method, which, although the study mentions that it values intra-operative variability, the method itself, being manual and on plaster models, allows inaccuracy.
- Being an observational retrospective study in study models it would have been very easy to have a control group to compare those same measures with patients without suspected HC.
- The study group was between the ages of 10 and 30. At ages close to 10 years, the upper canines have generally not erupted, nor have the second molars or even the premolars. Measures of intercanine or intermolar distance may vary between permanent and temporary dentition. This should be mentioned in the study. It could be a serious method error.
- The study does not mention whether the patient sample had active or passive condylar hyperplasia. There is a clear difference between a present metabolic activity assessed with a bone scan with growth already stopped.
- The study doesn't mention what kind of hyperplasia the patients had. It was Hemimandibular Elongation (horizontal vector), Hemimandibular Hyperplasia (vertical vector) or hybrid form. Authors such as Obwegeser *et al.* in 1984, Wolford *et al.* (2014) and Lopez *et al.* (2019), mention it adequately.
- The inclusion criteria mention that patients should have temporomandibular dysfunction within their diagnostic process. On the one hand, temporomandibular dysfunction is not a pathognomonic sign of the pathology. (There may be condylar hyperplasia without temporomandibular dysfunction). On the other hand, if this variable is taken, the occlusal findings could be the result of the dysfunction or hyperplasia? This should be mentioned in the discussion.
- In the results, the distance of the upper canines from the mid-palatal suture in women appears to be statistically significant. This finding could be related to the anatomical characteristics of female arches and not to arches of hyperplastic patients. This could be solved, using a control group.
- There are dental measures that could have been easily taken in the models and that would increase the variables of the study, such as: the angle classification, the torque of the posterior teeth.
- Finally, HC is an alteration that produces anatomical changes due to condylar overgrowth and positional changes due to the displacement of the jaw towards the contralateral side. Therefore, the long or hyperplastic side should be compared with the short or contralateral side in the variables taken.
- The conclusions postulate that asymmetric occlusal changes were observed in these patients, but the only statistically significant change was the superior canine distance in women. This should not be such a general conclusion and secondly this finding could be both occlusal and skeletal. 

Is the work clearly and accurately presented and does it cite the current literature?

Partly

If applicable, is the statistical analysis and its interpretation appropriate?

Yes

Are all the source data underlying the results available to ensure full reproducibility?

Partly

Is the study design appropriate and is the work technically sound?

No

Are the conclusions drawn adequately supported by the results?

Partly

Are sufficient details of methods and analysis provided to allow replication by others?

No

Reviewer Expertise:

Orthodontics, condylar hyperplasia, facial asymmetry.

I confirm that I have read this submission and believe that I have an appropriate level of expertise to confirm that it is of an acceptable scientific standard, however I have significant reservations, as outlined above.

# Response

**Published:** 2020-05-30

**Authors:** Ervina Sofyanti

**Affiliations:** Department of Orthodontics, Faculty of Dentistry, Indonesia

Dear Prof. Diego Fernando Lopez,

We thank the reviewer for the valuable comments and hope that the suggestions will improve the quality of our manuscript.

**Response to Reviewer:**

- **The authors make an investigation in which they observe characteristics in the dental arches of patients with suspected condylar hyperplasia (HC). Since they do not use a group of patients without suspected HC as a control group, they can only provide descriptive statistics and the only comparison they can make is between genders or according to age. Therefore, the title should mention " Differences in dental arch characteristics between genders in patients with suspected condylar hyperplasia in a North Sumatra subpopulation: a cross-sectional study ".**

We thank the reviewer for the suggestion. Previously we used the “evaluation” term to emphasize the characteristic of asymmetry and form of dental arch in CH case. According to the reviewer's suggestion, we have revised the title to “Differences in dental arch characteristics between genders in patients with suspected CH in a North Sumatra subpopulation”.

- **With respect to materials and methods, the authors use a millimeter rule to take measurements. There are now digital micrometric gauges as well as digital compasses to establish more accurate measurements that deliver decimals and thus provide measurements that may lead to statistically significant differences. (Measurements should be taken with these instruments that provide better accuracy). **

We thank the reviewer for the valuable comment. The measurement in this work was taken by millimeter ruler, we are adding measurement error in the manuscript: 

- Figure 1. Measuring angular upper arch asymmetry. The plaster cast model is positioned with a protractor at the cusp of the right upper arch canine (A); the protractor shows that PC of right upper arch canine is 46 ± 1° (B).

- Figure 2. Measuring angular lower arch asymmetry. The plaster cast model is positioned with a protractor at the cusp of the right lower canine (A); the protractor shows that PC of right lower arch canine is 39°±1° (B).

- **Additionally, the overlap of the upper palatal midline over the lower arch may be very inaccurate with manual techniques. At present, scanned models, tomography and the use of software reduce these errors in the method, which, although the study mentions that it values intra-operative variability, the method itself, being manual and on plaster models, allows inaccuracy.**

We thank the reviewer for the kind suggestion. The current measurement only gives the rough estimation/approximation to determine dental arch asymmetry and form, therefore accurate techniques in dental arch asymmetry including dimension in form and asymmetry analysis will be considered in near future. This statement will be mentioned in the discussion as limitation part of this study. In the manuscript: Although the procedure measurement was precise, using the millimeter ruler in analyzing arch form was a limitation in this study and should be revised with scanned models, tomography or the use of software in the further study.

- **Being an observational retrospective study in study models it would have been very easy to have a control group to compare those same measures with patients without suspected HC.**

We thank the reviewer for the advice. We decided to focus only on the suspected CH case, instead of control group due to determine the characteristics of dental arch asymmetry since we hypothesis that individual arch form based on race diversity, might present significant differences in the anatomic characteristics of the dental arch. Perhaps for our future study, we will conduct through detailed comparison with control group as suggested by the reviewer.

- **The study group was between the ages of 10 and 30. At ages close to 10 years, the upper canines have generally not erupted, nor have the second molars or even the premolars. Measures of intercanine or intermolar distance may vary between permanent and temporary dentition. This should be mentioned in the study. It could be a serious method error.**

We thank the reviewer for the comment. We will add the following sentence according to the reviewer's suggestion in the discussion. Most studies have reported that CH occurs between ages 10 and 30 years with abnormal growth of the hyperplasia cases. The hemimandibular hyperplasia occurs at significantly younger age. In the beginning, we include the sample with full erupted teeth until second molar in order to provide good measurement in this study. We want to apologize that we missed to input the meant age of this study that ranged from 18 to 27 year old and fulfilled the inclusion criterias.

- ** The study does not mention whether the patient sample had active or passive condylar hyperplasia. There is a clear difference between a present metabolic activity assessed with a bone scan with growth already stopped**.

We thank the reviewer for the suggestion. However we have mentioned that we used panoramic radiography due to in some emerging countries, like Indonesia, the CBCT recently progress then retrospective study found with limited radiograph. Nolte et al., 2015 was also mentioned about panoramic radiographs were suitable for screening CH, although the diagnostic standard was  to  assess the  condylar  growth  activity  using  bone  scintigraphy”

Therefore the following statement was incorporated into the manuscript:

The treatment objectives of previous report case mentioned that condylectomy to stop CH, surgery to correct skeletal discrepancy, and facelifts, augmentation  using  medpor  implants or fat grafts to achieve aesthetics  (Alyamani & Abuzinada, 2012; Lopez & Herrera-Guardiola, 2016; Lopez et al., 2017). The investigation of facial asymmetry  with CH complexity should be integrally planned during orthodontic treatment (Olate et al., 2013). Then, similar symptoms of glenoid fossa asymmetry and hemimandibular elongation that led to the difference in therapeutic approach and  suggested for careful correlation of clinical findings and imaging tests (Lopez & Ruiz, 2017).

- **The study doesn't mention what kind of hyperplasia the patients had. It was Hemimandibular Elongation (horizontal vector), Hemimandibular Hyperplasia (vertical vector) or hybrid form. Authors such as Obwegeser et al. in 1984, Wolford et al. (2014) and Lopez et al. (2019), mention it adequately.**

We thank the reviewer for the kind suggestion. Due to the certain limitations of radiography and since this was retrospective study led to our limitation in made the classification.

The references have been updated accordingly in the text:

Previous studies report several types of CH, based on radiographic and clinical characteristics that related to enlargement or hyperplasia of mandibular that will help the dental clinicians in understanding the complexity of facial asymmetry in 3 dimension (Obwegesser & Makek, 1986; Wolford et al., 2014; Lopez et al., 2019)

- **The inclusion criteria mention that patients should have temporomandibular dysfunction within their diagnostic process. On the one hand, temporomandibular dysfunction is not a pathognomonic sign of the pathology. (There may be condylar hyperplasia without temporomandibular dysfunction). On the other hand, if this variable is taken, the occlusal findings could be the result of the dysfunction or hyperplasia? This should be mentioned in the discussion.**

We thank the reviewer for the kind suggestion. Since the orthognatic surgery was not popular in our province, the treatment approach was only orthodontic treatment and stability of treatment was questioned. In this study, the similar symptoms make difficult, so that in my conclusion we suggested for dental arch evaluation in treating the orthodontic patients with suspected CH. We have revised by mentioning in discussion: “This findings related to the positive temporomandibular joint disorder as a concurrent symptoms with facial asymmetry, although this dysfunction is not a pathognomonic sign of the pathology.

- **In the results, the distance of the upper canines from the mid-palatal suture in women appears to be statistically significant. This finding could be related to the anatomical characteristics of female arches and not to arches of hyperplastic patients. This could be solved, using a control group.**

We thank the reviewer for the kind advice. We will consider about the control group in future study which related to asymmetry of glenoid fossa analysis as differential diagnosis for hemimandibular elongation (Lopez & Ruiz, 2017).

- **There are dental measures that could have been easily taken in the models and that would increase the variables of the study, such as: the angle classification, the torque of the posterior teeth.**

We thank the reviewer for the kind recommendation. Since these variables of the study will be fundamental for longitudinal study which is very important in treating the development of asymmetry occurred in CH. We have added this suggestion and will be considered in the future study. By understanding the characteristics of brackets, the clinician can obtain the desired teeth movement in achieve proper dental arch (Lopez et al., 2016).

- **Finally, HC is an alteration that produces anatomical changes due to condylar overgrowth and positional changes due to the displacement of the jaw towards the contralateral side. Therefore, the long or hyperplastic side should be compared with the short or contralateral side in the variables taken.**

We thank the reviewer for the comment. The following statements was added in the manuscript according to Lopez, 2017: “Previous studies reported Finishing  treatment  in the soft tissues usually require condylectomy to stop CH, surgery to correct skeletal discrepancy, and res correction, especially in  patients with longstanding condylar hyperplasia, and soft tissue procedures include facelifts, augmentation  using  medpor  implants or fat grafts to achieve aesthetics as alternative treatment in patients longstanding CH (Alyamani & Abuzinada, 2012; Lopez et al., 2017).

“Such as: The similar symptoms of glenoid fossa asymmetry and hemimandibular elongation that led to the difference in therapeutic approach and suggested for careful correlation of clinical findings and imaging tests (Lopez & Ruiz, 2017)

Thus, the characteristics of brackets should be well understood by the clinician to obtain the desired teeth movement in achieve proper dental arch (Lopez et al., 2016).

- **The conclusions postulate that asymmetric occlusal changes were observed in these patients, but the only statistically significant change was the superior canine distance in women. This should not be such a general conclusion and secondly this finding could be both occlusal and skeletal.**

We thank the reviewer for the suggestion. The following statement was incorporated in the conclusion of manuscript: “Indeed skeletal asymmetry, the evaluation of the dental arch characteristic symmetry and arch form showed asymmetric occlusal characteristics in orthodontics patient with suspected CH in North Sumatera subpopulation”.

# Peer review: Reviewer response for version 1

**DOI:** 10.5256/f1000research.25152.r62453

**Published:** 2020-05-12

**Authors:** Maarten J. Koudstaal

**Affiliations:** 1 The Dutch Craniofacial Centre, Department of Oral and Maxillofacial Surgery, Sophia’s Children’s Hospital Rotterdam, Erasmus University Medical Centre, Rotterdam, The Netherlands

This study aimed to evaluate dental arches by analyzing dental arch asymmetry and form in orthodontic patients with CH in a North Sumatra subpopulation. Retrospective study design in 34 patients, 10-30 years old.

 Abstract:

- Adequate.

 

 Introduction:

- Adequate.

Methods:

 Participants Inclusion:

- CH suspected subjects fulfilled the following characteristics: facial asymmetry based on photography, posterior cross bite and midline deviation in plaster casts analysis, and positive temporomandibular joint disorder in functional analysis.
- It is unclear if patients needed to have all of these? The reason for asking, a fair amount of patients with CH do not have any functional issues with the TMJ joint.
- At what time points were the records taken and studied? The same protocol for all patients? And at what ages?

Results:

- Age of inclusion? Would the authors expect a difference in the still active versus the non-active growth patients? If so please show the actual ages at inclusion and follow-up. And add a paragraph to the results about this.
- I do not see the patients classified as being either left or right side CH? Were they grouped? That does not make a lot of sense to me, especially when looking at the deviation of midline. Please elaborate.

 

 Discussion:

- Adequate

Tables:

- The legend should include explanation of the abbreviations used in the table.

Is the work clearly and accurately presented and does it cite the current literature?

Yes

If applicable, is the statistical analysis and its interpretation appropriate?

Not applicable

Are all the source data underlying the results available to ensure full reproducibility?

Yes

Is the study design appropriate and is the work technically sound?

Yes

Are the conclusions drawn adequately supported by the results?

Yes

Are sufficient details of methods and analysis provided to allow replication by others?

Yes

Reviewer Expertise:

NA

I confirm that I have read this submission and believe that I have an appropriate level of expertise to confirm that it is of an acceptable scientific standard.

# Response

**Published:** 2020-06-05

**Authors:** Ervina Sofyanti

**Affiliations:** Department of Orthodontics, Faculty of Dentistry, Indonesia

Dear Dr. Maarten J. Koudstaal,

We want to thank you for providing us with valuable advice for the manuscript.

 

- **CH suspected subjects fulfilled the following characteristics: facial asymmetry based on photography, posterior crossbite and midline deviation in plaster casts analysis, and positive temporomandibular joint disorder in functional analysis.**

We thank the reviewer for the suggestion. Since this is a study from a retrospective study of suspected CH patient's clinical records, we assessed that any pain, clicking, crepitation, pain, and other sign or symptom of positive temporomandibular joint disorder from the extraoral analysis will be included.

 

- **It is unclear if patients needed to have all of these? The reason for asking, a fair amount of patients with CH do not have any functional issues with the TMJ joint.**

We thank you for the comments. We include all these criteria in select cases based on patients' information medical records. We include patients with symptoms and/or TMD signs based on dental records.

 

- **At what time points were the records taken and studied? The same protocol for all patients? And at what ages? **

We thank you for kindly advising. The orthodontics patient's data medical records was collected and selected during January 2015 and March 2019. However, this information was collected by different orthodontic clinicians and the age of patients based on the date of birth stated in the medical record. We have also revised the resources data by adding the sample’s age. (Sofyanti E, Boel T, Satria D, et al.: Replication Data for Angular and linear measurement of the dental arch asymmetry and dental arch form. Harvard Dataverse, V1, UNF:6:GiTmDSqATBmhdCM366FFoQ== [fileUNF],2020. http://www.doi.org/10.7910/DVN/GONORA).

 

- **Results: Age of inclusion? Would the authors expect a difference in the still-active versus the non-active growth patients? If so please show the actual ages at inclusion and follow-up. And add a paragraph to the results about this. **

We thank you for the reviewer's advice. Based on the date of birth, the samples were selected between 10 to 30 years old in this study according to the previous study that reports about the age of condylar hyperplasia patients in the discussion phase. (Pacheco et al., 2010; Bharathi et al., 2014; Lopez & Herrera-Guadiola., 2016; Lopez et al., 2017; Goulart et al., 2018). The limitation of this study was less information about the activity of condylar growth due to the dental hospital didn/t have 3D CBCT in that time to evaluate the condylar precisely.

- **I do not see the patients classified as being either left or right side CH? Were they grouped? That does not make a lot of sense to me, especially when looking at the deviation of the midline. Please elaborate.**

Based on previous medical records, we include samples with a deviation of midline in occlusion transversally. In this study, we analyzed the upper and lower arch based on Scanavini and Raberin method in the plaster cast of suspected CH as mentioned in the material and method

based on previous studies about CH clinical findings.
